# Cautionary note on contamination of reagents used for molecular detection of SARS-CoV-2

**DOI:** 10.1093/clinchem/hvaa214

**Published:** 2020-09-07

**Authors:** Jim F Huggett, Vladimir Benes, Stephen A Bustin, Jeremy A Garson, Karthyn Harris, Martin Kammel, Mikael Kubista, Timothy D McHugh, Jacob Moran-Gilad, Tania Nolan, Michael W Pfaffl, Marc Salit, Greg Shipley, Peter M Vallone, Jo Vandesompele, Carl Wittwer, Heinz Zeichhardt

**Affiliations:** 1 National Measurement Laboratory (NML) at LGC, Queens Rd, Teddington, TW11 0LY, United Kingdom; 2 School of Biosciences & Medicine, Faculty of Health & Medical Science, University of Surrey, Guildford, GU2 7XH, United Kingdom; 3 Genomics Core Facility, EMBL Heidelberg, Heidelberg, Germany; 4 Medical Technologies Research Centre, Anglia Ruskin University, Cambridge and ChelmsfordGenomics Core Facility, EMBL Heidelberg, Heidelberg, Germany; 5 Division of Infection and Immunity, University College London, London, UK; 6 Microbiology Department, Great Ormond Street Hospital NHS Foundation Trust, London, UK; 7 INSTAND e.V., Duesseldorf; IQVD GmbH, Institut fuer Qualitaetssicherung in der Virusdiagnostik, Berlin, Germany; 8 TATAA Biocenter, Sweden and Institute of Biotechnology of the Czech Academy of Sciences; 9 Center for Clinical Microbiology, Division of Infection and Immunity, University College London, United Kingdom; 10 Department of Health Systems Management, School of Public Health, Faculty of Health Sciences, Ben Gurion University of the Negev, Beer Sheva, Israel; 11 Institute of Population Health, Faculty of Medical and Human Sciences, University of Manchester, UK, The Gene Team, UK; 12 Animal Physiology and Immunology, Technical University of Munich, School of Life Sciences Weihenstephan, Freising, Germany; 13 Joint Initiative for Metrology in Biology, SLAC National Accelerator Laboratory, Menlo Park, CA, USA; 14 Departments of Bioengineering and Pathology, Stanford University, Stanford, CA, USA; 15 Shipley Consulting, LLC, Vancouver, WA, USA; 16 National Institute of Standards and Technology, 100 Bureau Dr, Gaithersburg, MD, USA; 17 Department of Biomolecular Medicine, Ghent University; 18 Biogazelle, Belgium; Ghent University, Belgium; 19 Department of Pathology, University of Utah, 30 N 1900 E, Salt Lake City, UT, USA; 20 INSTAND e.V., Duesseldorf; Charité – University Medicine Berlin; IQVD GmbH, Institut fuer Qualitaetssicherung in der Virusdiagnostik, Berlin, Germany

**Keywords:** SARS-CoV-2, RT-qPCR, Contamination, False positive, Molecular Diagnosis

## Abstract

Reverse transcription (RT)-PCR, the principal diagnostic method applied in the world-wide struggle against COVID-19, is capable of detecting a single molecule of a viral genome. Correctly designed and practiced RT-PCR assays for SARS-CoV-2 should not cross react with similar but distinct viral pathogens, such as the coronaviruses associated with the common cold, and should perform with very high analytical sensitivity. This analytical performance is predicated on the ability of the method to detect the presence of the selected nucleic acid target, without detection of a false positive signal.

Unlike many other diagnostic methods, such as ELISA, there should be no "blank" signal in RT-PCR diagnosis of SARS-CoV-2. False positive results may occur during testing, but should not be considered as a background signal or factored into specificity calculations. Like false negative results, it is incumbent on laboratory practitioners to be wary of, and monitor for, false positives. The handful of reports of "background" SARS-CoV-2 signal *(1, 2)* are unlikely to be due to primer artefacts or cross reactivity with other pathogens, or human template, given that the assays in question are referenced by the World Health Organization *(3)* and have been used across the globe without such observation. The only practical or technical source of so-called 'background' for an optimally designed SARS-CoV-2 diagnostic assay is contamination, which is the main source of false positives when conducting any PCR test.

There are two principal contamination routes: cross-contamination between specimens or synthetically derived target nucleic acids. Cross-contamination from a positive clinical sample to a negative one can occur during specimen sampling, handling, processing or analysis. While this risk is substantial for SARS-CoV-2, due to potentially high viral loads, it is not background but instead a variable technical artefact.

Synthetically derived PCR amplicon contamination can arise from the billions of copies of the molecule of interest generated in the course of a PCR assay. Without proper care these reaction products can contaminate samples or reagents, becoming false positives in subsequent tests. PCR

practitioners have long known of the risk of carry-over contamination and have devised procedures and laboratory measures to minimize it *(4, 5)*. Yet poor understanding of this artefact has led to erroneous, and sometimes tragic, claims such as reported false evidence linking measles, mumps and rubella (MMR) vaccine with autism *(6)*.

There is another source of synthetically derived contamination that may be particularly relevant to SARS-CoV-2 testing. A common practice for PCR assay development is for the developer to commission the synthesis of the intended DNA target, using phosphoramadite chemistry, which is a globally established process offered by a number of manufacturers, as a positive control. The synthesis of these gene fragments is typically at nanomole scale and will produce in excess of a thousand trillion ($10^{15}$) copies of single stranded DNA. It is an essential practice to assure that this control template is made at different sites, usually from alternate vendors, from those sites making the other PCR reagents, to avoid this major potential source of contamination. However, as the number of laboratories developing assays and reference material for the global SARS-CoV-2 pandemic is unprecedented, selecting different vendors may no longer prevent this source of contamination.

There are already examples of such assay-derived contamination occurring *(7)* that has hampered the diagnostic response to COVID-19 *(8)*, with RT-PCR reagents becoming contaminated regardless of whether they are used to detect SARS-Cov-2 (Figure 1). This level of production of synthetic template has the potential to not only generate false-positives and indirectly to reduce the sensitivity of our principal diagnostic method, but it may also limit other areas of research such as measuring viral spread using environmental sources such as wastewater *(9)*.

With the worldwide application of RT-PCR to a handful of the same conserved viral genes, we fear that a quotidian source of contamination of SARS-CoV-2 diagnostic RT-PCR is being experienced, yet overlooked. Some of the laboratories applying the procedure may be unaware that such contamination may compromise the accuracy of the very methods we are currently depending on to monitor this pandemic. In response, there are basic steps users can apply to monitor and reduce contamination (Box 1). While synthesis of molecular targets will remain an important tool for assay development, vendors and users may ask whether, given the vast amount of SARS-CoV-2 sequence that has already been made, it is possible for template to be obtained using collaborative or commercial sources other than chemical synthesis. Should synthesis still be required, vendors could explore solutions, like incorporating 'watermarks' *(10)* into the synthesized material, to allow these sources of positive signal to be distinguished from actual SAR-CoV-2 RNA.

A timely global response to this pandemic has been made possible by RT-PCR. To fully exploit the sensitivity of this method, we must be cognizant of and rigorously test for potential contamination of reagents. As with the pandemic, knowledge of and testing for contamination will prevent it from spreading. Moreover, lessons learnt with respect to this emerging global challenge of reagent contamination should be taken into consideration, in preparedness and response planning for future pandemics.

**Disclaimer**

The opinions, recommendations, findings, and conclusions in this publication do not necessarily reflect the views or policies of NIST or the United States Government.

**Author Contributions:** *All authors confirmed they have contributed to the intellectual content of this paper and have met the following 4 requirements: (a) significant contributions to the conception and design, acquisition of data, or analysis and interpretation of data; (b) drafting or revising the article for intellectual content; (c) final approval of the published article; and (d) agreement to be accountable for all aspects of the article thus ensuring that questions related to the accuracy or integrity of any part of the article are appropriately investigated and resolved.*

**Authors' Disclosures or Potential Conflicts of Interest:** *Upon manuscript submission, all authors completed the author disclosure form. Disclosures and/or potential conflicts of interest:*

**Employment or Leadership:** M. Kubista, Tataa Biocenter; J. Moran-Gilad, ESCMID; C. Wittwer, *Clinical Chemistry*, AACC.
**Consultant or Advisory Role:** J. Moran-Gilad, Ella Therapeutics Inc.; H. Zeichhardt, Gesellschaft für Virologie eV.
**Stock Ownership:** M. Kubista, Tataa Biocenter; H. Zeichhardt, Gesellschaft für Biotechnologische Diagnostik mbH.
**Honoraria:** None declared.
**Research Funding:** This work was supported by the UK National Measurement System and the European Metrology Programme for Innovation and Research (EMPIR) joint research project [18HLT03] "SEPTIMET" (which has received funding from the EMPIR programme co-financed by the Participating States and the European Union's Horizon 2020 research and innovation programme) and supported by the DOE Office of Science through the National Virtual Biotechnology Laboratory, a consortium of DOE national laboratories focused on response to COVID-19, with funding provided by the Coronavirus CARES Act. M. Kubista, RVO 86652036 BIOLEV L2.1.05/1.1.00/02.0109; M. Salit, US DOE National Virtual Biotechnology Laboratory.
**Expert Testimony:** None declared.
**Patents:** None declared.

Figure 1.
RNA extracts from 60 SARS-CoV-2 negative clinical samples (nasopharyngeal swabs and aspirates) and a positive control (RNA transcript of the SARS-CoV-2 nucleocapsid (N) gene) were amplified in parallel in two multiplexed reactions: A) amplification plot showing SARS-CoV-2 fluorescence from a duplex reaction that contains SARS-CoV-2 and RNaseP primers and probes. B) amplification plot of SARS-CoV-2 fluorescence in a triplex PCR assay including the targets SARS-CoV-2, RNaseP, and an internal spike positive control (phocine distemper virus, PDV). This illustrates SARS-CoV-2 target contamination from a non SARS-CoV-2 assay, in this case PDV: half of the negative patient samples now test positive for SARS-CoV-2. The real-time amplification plots for SARS-CoV-2 (N2) were performed on a QuantStudio 5 thermal cycler (Thermo Fisher) using the One Step PrimeScript III RT-PCR Kit (Takara). X axis = PCR cycles, Y axis = Fluorescence, curved lines = plots of amplified SARS-CoV-2 target.

**A**

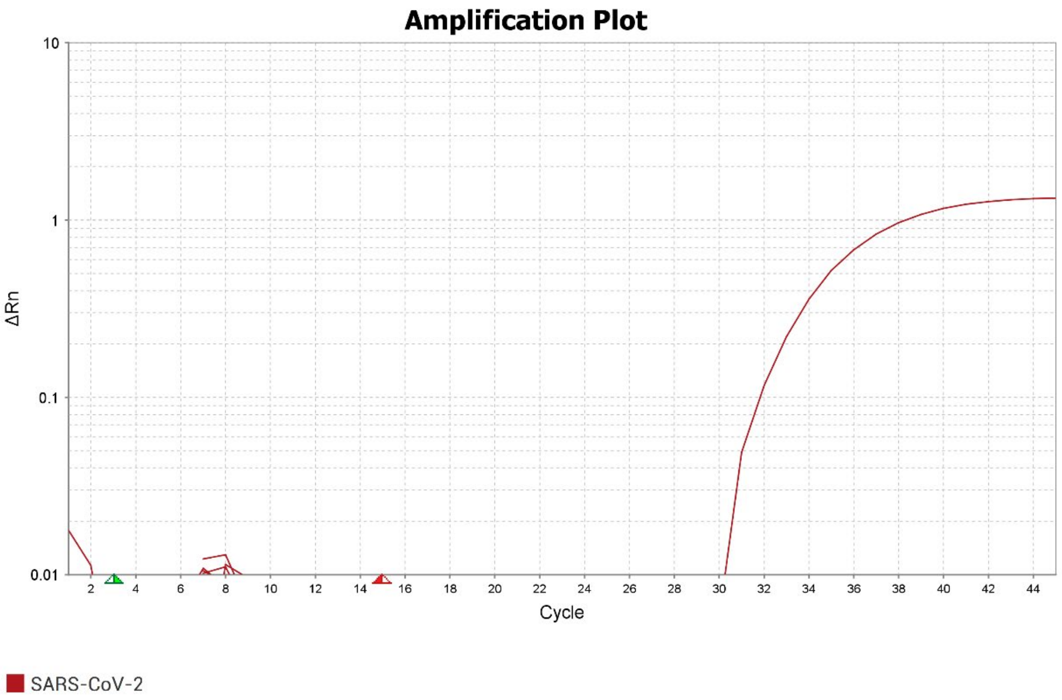

Result: 60 negative patients

**B**

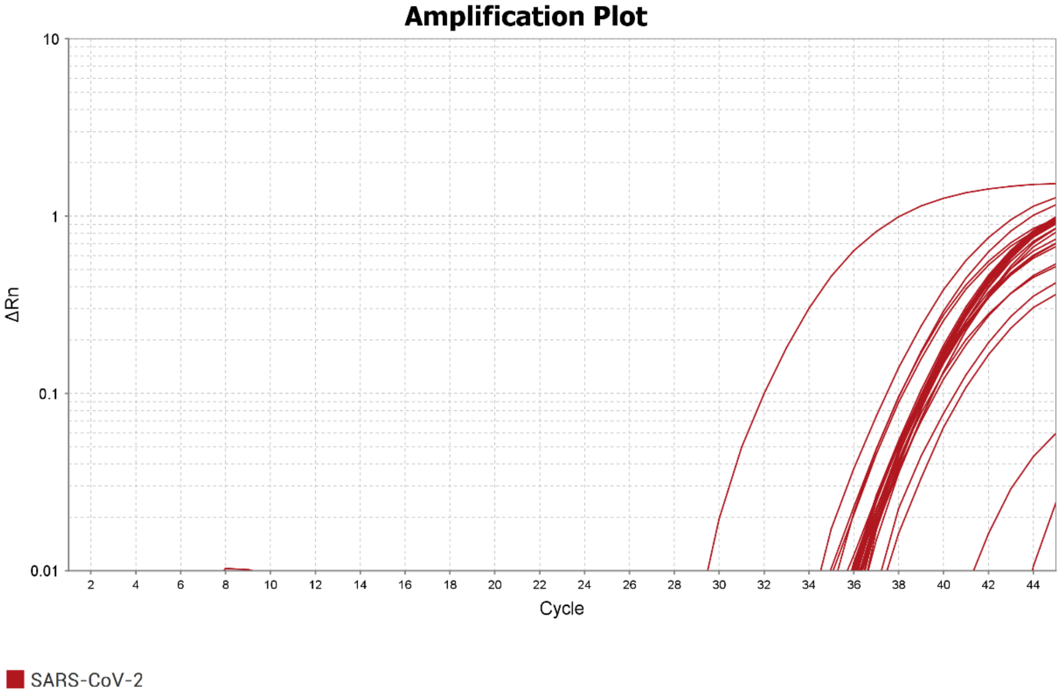

Result: 30 negative patients & 30 false positive patients

Box 1. How to be confident your SARS-CoV-2 results are not corrupted with contamination

**Test for it**

- Assume reagents may contain contamination. Quality control reagents prior to their use (primers, probes, PCR mastermix, water) using multiple negative control replicates alongside a positive control. 10 negative controls in a 96 well plate represents a practical number, however larger numbers of replicates will better assure confidence in ruling out low-level contamination, which can appear both stochastically and infrequently.
- Aliquot reagents for single time use, especially nuclease-free water.
- Implement control procedures that include extraction blanks that contain carrier RNA; the latter (present in negative patient extracts) is important for measuring low level contamination. Consider using multiple extraction blanks distributed amongst sample reactions to detect low level contamination.
- Further information on the precise source of contamination can be provided by including reverse transcription negative reactions; this will confirm DNA and not viral RNA as the source.

**Apply caution when results are close to the limit of detection of assay**

- Beware of large numbers of results with high Cq values near the assay limit of detection.
- Consider the pattern of results. If low signal positives are not randomly distributed (e.g. if they occur adjacent to a high titre sample) this suggest sample cross-contamination. Consider repeating such low positive samples.
- Consider influences of pre-analysis and sample cross-contamination.
- If possible, test for more than one SARS-CoV-2 target gene.

**Take preventive measures**

- Physically separate PCR setup and sample handling steps (and equipment) from those used for PCR analysis. It is absolutely crucial to use pre- and post-PCR rooms as well as unidirectional transit from pre to post-PCR laboratories
- Consider steps during preparation that may lead to contamination through aerosol production: pipetting (high throughput), centrifuges, etc. may lead to aerosols that can result in cross-contamination.

**Get rid of it**

- Discard all reagents linked to contaminated reactions. While systematic evaluation may determine which reaction component is the culprit, it is recommended to start from scratch and replace all the reagents.
- Deep clean the laboratory using proven solutions that destroy nucleic acids (e.g. bleach and UV) on a daily basis
- If contamination persists, users may need to halt clinical testing and redesign the assay to different part of the pathogen's genome.
